# Characteristics of the Bacterial Community in Alpine Meadows in Response to Altitude and Aspect in the Qilian Mountains, Northwest China

**DOI:** 10.1002/ece3.70769

**Published:** 2025-01-08

**Authors:** Qiang Li, Ying Zhang, Xiaoni Liu, Fan Yang

**Affiliations:** ^1^ State Key Laboratory of Plateau Ecology and Agriculture/College of Agriculture and Animal Husbandry Qinghai University Xining China; ^2^ College of Grassland Science Gansu Agricultural University Lanzhou China

**Keywords:** FAPROTAX function, Qinghai‐Tibetan Plateau, soil bacterial communities, topographic factors

## Abstract

As one of the most sensitive and fragile alpine ecosystems in the Qilian Mountains, the alpine meadow holds significant scientific importance in understanding the changes in the characteristics of soil bacterial community in response to altitude and aspect variations. In our study, we analyzed the composition, diversity, and function of soil bacterial communities in alpine meadows at different altitudes and aspects and their relationship with environmental factors. Our results indicate that altitude and aspect orientation significantly influences the diversity index and composition of soil bacterial communities. Specifically, the Shannon and Chao1 indices of soil bacteria initially increased and then decreased with increasing altitude, with the Shannon index being lower in shady aspects compared to sunny aspects, and the Chao1 index being lower in sunny aspects above an altitude of 3400 m compared to shady aspects at the exact altitudes. Using Linear Discriminant Analysis Effect Size analysis, we identified 77 bacterial species in the research area, with key ecological functions primarily associated with nitrification, aerobic ammonia oxidation, and chitinolysis. Furthermore, we found that soil water content and Urease were the main factors influencing bacterial community composition. Our findings underscore the significant impact of altitude orientation on bacterial communities in alpine grasslands, emphasizing the importance of considering bacterial differences in evaluating alpine grassland health.

## Graphical abstract

Soil bacterial communities composition and diversity in alpine meadows were significant differences at different altitudes and aspect. TN and Urease were the main factors affecting the bacterial community. Altitude and aspect changed the composition and diversity of bacterial.

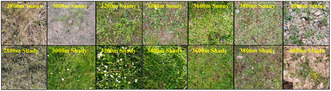

## 1 Introduction

Mountain ecosystems constitute a significant component of terrestrial ecosystems and harbor unique ecological functions (Li et al. 2018; Li et al. 2022a, 2022b). Altitude and aspects within mountain ecosystems bring about substantial changes in plants, soil, and climate, among other factors, thereby exerting further influence on microbial communities. In northwest China, the Qilian Mountains act as a natural barrier and transition zone between the Qinghai‐Tibet Plateau, the Loess Plateau, and the Mongolian Plateau (Li, Liu et al. 2021; Li, Qiu et al. 2021; Li, Yang et al. 2021). Due to its geographical location, the Qilian Mountains region is highly sensitive to global climate change (Li et al. 2022a, 2022b). As a predominant vegetation type in the area, Alpine meadows play a crucial role in maintaining ecological stability and health (Chen et al. 2023). Scholars have conducted comprehensive research on vegetation characteristics, and soil physical and chemical properties across different altitudes and aspects (Li, Liu et al. 2021; Li, Qiu et al. 2021; Li, Yang et al. 2021). Vegetation composition and soil nutrient content in alpine meadows undergo regular changes with variations in altitude and aspect, inevitably influencing soil bacterial communities (Zhan et al. 2018; Jing et al. 2014). However, despite being a significant driver of ecosystem dynamics, relatively few studies have explored the changes in soil bacterial communities with altitude and aspect. Therefore, investigating the mechanisms underlying the alteration of soil bacterial communities in mountain ecosystems is imperative for understanding the distribution patterns of terrain factors in altitude and aspect within grasslands.

As the most abundant microbial group, soil bacteria are paramount decomposers of soil organic matter and litter, playing a pivotal role in regulating biogeochemical cycles and driving energy flow and material cycles in the biosphere (Liu et al. 2023; Wu et al. 2023; Chen et al. 2016; Fang et al. 2018). Shen et al. (2015) and Deng et al. (2016) have demonstrated that environmental changes significantly influence bacterial communities, making them crucial indicators for evaluating ecosystem function and material cycling. Altitude and aspect, as forms of geographical environmental change, can lead to variations in environmental indices (Korner 2007). Changes in soil bacterial communities are triggered by alterations in soil nutrients, temperature, humidity, and aboveground vegetation (Li et al. 2016; Sun et al. 2021). With the rapid development of high‐throughput sequencing technology, studies have documented variations in soil bacterial diversity and community structure along altitude gradients. For instance, as altitude increases, soil bacterial diversity exhibits declining concave, hump, and stepped distribution patterns (Nottingham et al. 2018; Shen et al. 2020; Li et al. 2018). However, there have been relatively few investigations into changes in soil bacterial diversity and community structure under different aspects. The function of the soil bacterial ecosystem is closely tied to its community structure characteristics (Li et al. 2022a, 2022b). Shen et al. (2016) demonstrated that the abundance of functional genes of soil bacteria in Changbai Mountain increases with altitude, while soil bacterial metabolic pathway genes undergo significant changes along altitude gradients in Sejila Mountain (An et al. 2021). These studies have focused on altitude gradients with significant variations in different vegetation and ecosystems. In contrast, the distribution of bacterial community characteristics within a single ecosystem across altitudes and aspects remains largely unexplored. Furthermore, the relationship between soil bacterial community structure and function remains unclear. Therefore, by selecting the same vegetation type and investigating the response of bacterial communities to altitude and aspect, it will be possible to more accurately assess the impact of changes in environmental factors on bacterial community characteristics and elucidate their driving mechanisms.

In this paper, we propose the scientific hypotheses: whether topographic factors significantly alter soil bacterial and there is a relationship between soil bacterial communities and environmental factors. Based on these hypotheses, we analyzed the composition, diversity, and functional characteristics of the soil bacterial community in alpine meadows across different altitudes and aspects in the Qilian Mountains. We also explored the key environmental factors affecting the bacterial community. This research aims to shed light on the structure and function of microbial communities in alpine mountains under the influence of climate change and anthropogenic disturbances.

## 2 Materials and Methods

### 2.1 Research Area

The research site is situated in the Jinqiang River Basin (E102.442°–102.917°, N37.123°–37.298°) within the eastern Qilian Mountains of China (Li et al. 2022a, 2022b). Altitude and annual average temperature range from 2600 to 4300 m and −0.1°C to 0.6°C, respectively. Annual rainfall was between 400 and 480 mm and the accumulated temperature ≥ 0°C approximately 1360°C, while average annual evaporation is about 1153 mm (Li et al. 2022a, 2022b). The average relative humidity is 55%. The primary grassland types include alpine meadows, alpine grasslands, and temperate desert grasslands, with subalpine meadows and mountain meadow soil being predominant (Li et al. 2022a, 2022b). Soil water content (SWC) ranges from 50% to 80%, while pH levels range from 6.94 to 8.17 (Li et al. 2022a, 2022b).

### 2.2 Plot Setting

**FIGURE 1:**
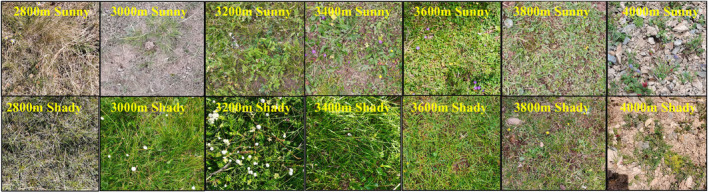
Landscape photos of the sampling sites.

From July 28 to August 5, 2020, we selected seven altitudes (2800, 3000, 3200, 3400, 3600, 3800, and 4000 m) in the study area. At each altitude, we designated two aspects: sunny and shady. Three 10 m × 10 m sampling strips were chosen for each treatment, as illustrated in Figure 1 (Li et al. 2022a, 2022b).

Within each sampling area, we established three 50 cm × 50 cm quadrats along the diagonal (three replicates), focusing on measuring vegetation characteristics (Table 1; Li et al. 2022a, 2022b). After collecting plant samples, we collected corresponding surface soil samples from the 0–30 cm depth, divided into two parts. The first part was utilized to assess soil physical and chemical properties (stored in a soil collection box at room temperature; Table 1; Li et al. 2022a, 2022b). The second part was preserved for soil bacterial community analysis and stored in a − 80°C dry ice soil collection box.

**TABLE 1:** Basic information of alpine meadow in different latitude and aspects (Li, Liu, et al. 2021; Li, Qiu, et al. 2021; Li, Yang, et al. 2021).

Altitude m	Aspect	Latitude and longitude	Coverage (%)	GLH (cm)	AGB (g m^−2^)	SBD (g cm^−3^)	SWC (%)	STP (%)	SOC (g kg^−1^)	TN (g kg^−1^)	TP (g kg^−1^)	AP (mg kg^−1^)	AK (mg kg^−1^)	Urease (mg g^−1^ 24 h^−1^)	Catalase (mL [0.1 N Kmno4)] h^−1^ g^−1^)	ALP (mg g^−1^ 24 h^−1^)	Sucrase (mg g^−1^ 24 h^−1^)
2800	Sunny	E102.9107°, N37.1467°	46.67 ± <2.89Bd	7.07 ± <0.57Ac	106.67 ± <14.10Bc	0.96 ± <0.04Ab	19.07 ± <4.66Ae	51.92 ± <2.58Ac	34.12 ± <5.79Ac	1.47 ± <0.12Ac	2.56 ± <0.31Aa	14.45 ± <3.49Ab	187.26 ± <24.53Ac	11.5 ± <0.5Aa	44.90 ± <1.37Aa	0.16 ± <0.09Ac	16.9 ± <2.5Ab
	Shady	E102.9168°, N37.1230°	66.67 ± <2.89Ad	8.11 ± <0.57Ac	142.13 ± <18.11Ac	0.87 ± <0.05Ac	25.30 ± <3.90Ad	60.85 ± <1.48Bc	59.42 ± <9.18Bd	3.44 ± <0.48Be	2.42 ± <0.50Aa	17.82 ± <5.08Abc	157.63 ± <13.94Ac	10.4 ± <0.3Ba	42.18 ± <0.63Ba	0.42 ± <0.06Bb	28.4 ± <4.6Ba
3000	Sunny	E102.7830°, N37.2100°	70.00 ± <2.89Bb	8.95 ± <0.51Ab	186.67 ± <13.28Bb	0.77 ± <0.06Acd	21.95 ± <3.16Bd	64.80 ± <1.93Ab	66.73 ± <5.58Ab	3.91 ± <0.19Ab	1.77 ± <0.39Ab	15.26 ± <4.42Ab	209.36 ± <34.04Abc	12.2 ± <0.2Aa	43.79 ± <1.52Aa	0.49 ± <0.06Ab	21.7 ± <4.8Aab
	Shady	E102.8056°, N37.1705°	83.33 ± <5.00Ab	9.88 ± <0.76Ab	219.47 ± <15.56Ab	0.70 ± <0.02Ad	34.02 ± <2.76Ad	66.40 ± <2.23Aab	82.06 ± <3.81Bb	5.19 ± <0.54Bc	1.50 ± <0.36Ab	19.88 ± <3.70Ab	191.77 ± <13.81Ab	7.4 ± <0.4Bb	38.65 ± <1.77Bb	0.51 ± <0.10Aab	28.6 ± <4.2Aa
3200	Sunny	E102.7441°, N37.2274°	80.00 ± <5.00Ba	11.19 ± <0.48Ba	214.13 ± <21.09Ba	0.68 ± <0.02Ad	31.02 ± <3.56Ba	67.80 ± <2.00Aa	85.66 ± <10.08Aa	5.80 ± <0.64Aa	1.59 ± <0.22Ab	24.62 ± <2.50Aa	286.61 ± <9.34Aa	8.3 ± <0.2Ab	39.41 ± <0.64Ab	0.60 ± <0.10Aa	22.3 ± <4.1Aab
	Shady	E102.7096°, N37.1960°	96.67 ± <2.89Aa	13.04 ± <0.56Aa	307.60 ± <32.56Aa	0.66 ± <0.02Ad	64.36 ± <3.89Ac	72.92 ± <3.10Aa	99.26 ± <8.21Aa	6.34 ± <0.18Aa	1.42 ± <0.26Ab	28.75 ± <2.65Aa	221.06 ± <8.92Ba	4.9 ± <0.9Bc	35.98 ± <0.74Bb	0.67 ± <0.02Aa	27.4 ± <5.0Aa
3400	Sunny	E102.5282°, N37.2649°	73.33 ± <2.89Bb	7.40 ± <0.25Bc	177.47 ± <13.86Ab	0.79 ± <0.04Ac	38.91 ± <1.56Bb	61.17 ± <2.60Abc	76.48 ± <10.91Aab	4.91 ± <0.45Aab	1.52 ± <0.27Abc	13.00 ± <3.06Abc	246.16 ± <15.83Ab	3.9 ± <0.3Ac	32.49 ± <1.34Ac	0.37 ± <0.07Ab	28.5 ± <1.9Aa
	Shady	E102.6265°, N37.2191°	80.00 ± <2.89Abc	9.70 ± <0.49Ab	200.27 ± <14.62Ab	0.68 ± <0.02Bd	57.13 ± <2.03Ab	63.92 ± <3.32Ab	70.03 ± <7.21Ac	4.58 ± <0.39Ab	1.12 ± <0.21Ab	17.03 ± <2.17Ab	167.56 ± <27.77Bbc	2.1 ± <0.5Bd	22.38 ± <1.78Bc	0.47 ± <0.11Ab	23.2 ± <1.8Bab
3600	Sunny	E102.4623°, N37.2797°	70.00 ± <5.00Ab	5.40 ± <0.59Bd	113.74 ± <9.23Bc	0.82 ± <0.03Ac	52.18 ± <2.00Abc	58.52 ± <0.92Abc	71.60 ± <7.11Aab	4.29 ± <0.26Ab	1.23 ± <0.32Abc	11.41 ± <3.58Abc	152.90 ± <26.17Acd	2.5 ± <0.2Ad	33.14 ± <1.28Ac	0.22 ± <0.11Abc	26.7 ± <4.0Aa
	Shady	E102.5771°, N37.2176°	75.00 ± <2.89Ac	7.77 ± <0.51Ac	150.80 ± <7.08Ac	0.80 ± <0.05Ac	55.42 ± <5.66Aa	61.72 ± <5.42Ab	65.45 ± <8.46Ac	3.65 ± <0.38Bc	1.07 ± <0.32Ab	13.86 ± <3.23Abc	154.27 ± <6.79Ac	1.5 ± <0.4Bde	16.98 ± <1.73Bd	0.41 ± <0.01Bb	16.7 ± <4.4Bb
3800	Sunny	E102.4310°, N37.2939°	60.00 ± <2.89Ac	4.68 ± <0.19Be	70.14 ± <8.52Ad	0.97 ± <0.02Ab	37.54 ± <4.40Bc	51.92 ± <3.55Ac	54.85 ± <5.20Ac	3.17 ± <0.25Ad	0.77 ± <0.15Ad	10.85 ± <2.11Abc	128.24 ± <18.27Ad	1.9 ± <0.9Ade	23.64 ± <1.11Ad	0.33 ± <0.07Abc	26.8 ± <2.8Aa
	Shady	E102.5457°, N37.2157°	65.00 ± <2.89Ad	6.96 ± <1.06Ac	78.937 ± <15.60Ad	0.96 ± <0.04Ab	48.66 ± <4.53Abc	61.03 ± <1.33Bc	59.21 ± <5.96Ac	1.49 ± <0.22Bd	0.54 ± <0.12Ac	12.92 ± <1.22Ab	118.30 ± <12.75Ad	1.4 ± <0.7Ade	16.74 ± <0.82Bd	0.27 ± <0.06Bc	10.5 ± <1.3Ac
4000	Sunny	E102.4417°, N37.2983°	25.00 ± <5.00Ae	3.66 ± <0.08Af	56.14 ± <21.53Ad	1.10 ± <0.05Aa	20.16 ± <2.42Bd	33.20 ± <3.37Ac	18.98 ± <6.10Ae	0.67 ± <0.22Af	1.15 ± <0.19Ab	5.50 ± <1.67Ac	103.01 ± <10.00Ad	1.0 ± <0.1Ae	20.06 ± <1.21Ae	0.42 ± <0.07Ab	9.3 ± <2.5c
	Shady	E102.5383°, N37.2039°	20.00 ± <1.15Ae	4.06 ± <0.48Ad	66.00 ± <17.21Ad	1.03 ± <0.02Aa	40.26 ± <2.91Ad	59.47 ± <2.25Bd	15.91 ± <2.17Ad	0.35 ± <0.05Be	1.28 ± <0.12Ac	8.63 ± <1.18Bc	109.09 ± <13.00Ad	0.3 ± <0.2Be	11.53 ± <0.87Be	0.20 ± <0.03Bc	7.4 ± <1.2d

*Note:* The lowercase letters indicate the difference between the same aspect and different altitudes (*p* ﹤ 0.05), and the capital letters indicate the difference between the same altitude and different aspect (*p* ﹤ 0.05).

Abbreviations: AGB, above‐ground biomass; AK, available potassium; ALP, alkaline phosphatase; AP, available phosphorus; GLH, grass layer height; SBD, soil bulk density; SOC, soil organic carbon; STP, soil total porosity; SWC, soil water content; TC, total coverage; TN, total nitrogen.

### 2.3 Soil Bacteria DNA Extraction and Amplification and Library Construction

Soil content samples were immediately snap‐frozen and stored at −80°C upon collection. Bacterial DNA was extracted from the soil using the DNeasy PowerSoil kit (Qiagen, Hilden, Germany) following the manufacturer's instructions. The concentration and integrity of DNA were assessed using a NanoDrop 2000 spectrophotometer (Thermo Fisher Scientific, Waltham, MA) and agarose gel electrophoresis, respectively. Polymerase chain reaction (PCR) amplification of the V3–V4 hypervariable regions of the bacterial 16S rRNA gene was conducted in a 25 μL reaction utilizing universal primer pairs (5′‐TACGGRAGGCAGCAG‐3′). The reverse primer contained a sample barcode, and both primers were linked with an Illumina sequencing adapter.

Amplicon quality was assessed through gel electrophoresis. PCR products were purified using Agencourt AMPure XP beads (Beckman Coulter Co.) and quantified with a Qubit doube stranded DNA assay kit. Subsequently, the concentrations were adjusted for sequencing. Sequencing was performed on an Illumina NovaSeq6000 platform, generating two paired‐end read cycles of 250 bases each (Illumina Inc., San Diego, CA; OE Biotech Company, Shanghai, China).

### 2.4 Data Processing and Analysis

The data were organized using Excel 2019, and all values are presented as mean ± standard error. One‐way analysis of variance and significance tests were conducted using Statistical Package for Social Sciences 21.0. Canoco 5.0 software was utilized for redundancy analysis (RDA) and Monte Carlo tests (Data normalization was performed before RDA analysis). Raw sequencing data were provided in FASTQ format. Paired‐end reads underwent preprocessing using cutadapt software to detect and remove adapters. Following trimming, low‐quality sequences were filtered out, denoised merged, and chimeric reads were detected and removed using DADA2 with default parameters in QIIME2. Subsequently, the software generated representative reads and the ASV abundance table. The representative read of each ASV was selected using the QIIME 2 package. All representative reads were annotated and blasted against the Silva database Version 138 (16S) using a q2‐feature‐classifier with default parameters. Microbial diversity in soil content samples was assessed using alpha diversity metrics, including the Chao1 and Shannon indexes. Unweighted Unifrac Principal Coordinates Analysis and phylogenetic tree construction were conducted using the Unifrac distance matrix generated by QIIME software. The 16S rRNA gene amplicon sequencing and subsequent analysis were performed by OE Biotech Co. Ltd., (Shanghai, China). FAPROTAX software provides a functional classification database based on species information, encompassing over 80 functional classifications such as carbon (C), nitrogen (N), phosphorus, and sulfur cycles and others like animal–plant pathogens, methane production, and fermentation. This database covers more than 4600 prokaryotic species and effectively predicts the biochemical cycle processes of environmental samples.

## 3 Results

### 3.1 Soil Bacterial Community Diversity

**FIGURE 2:**
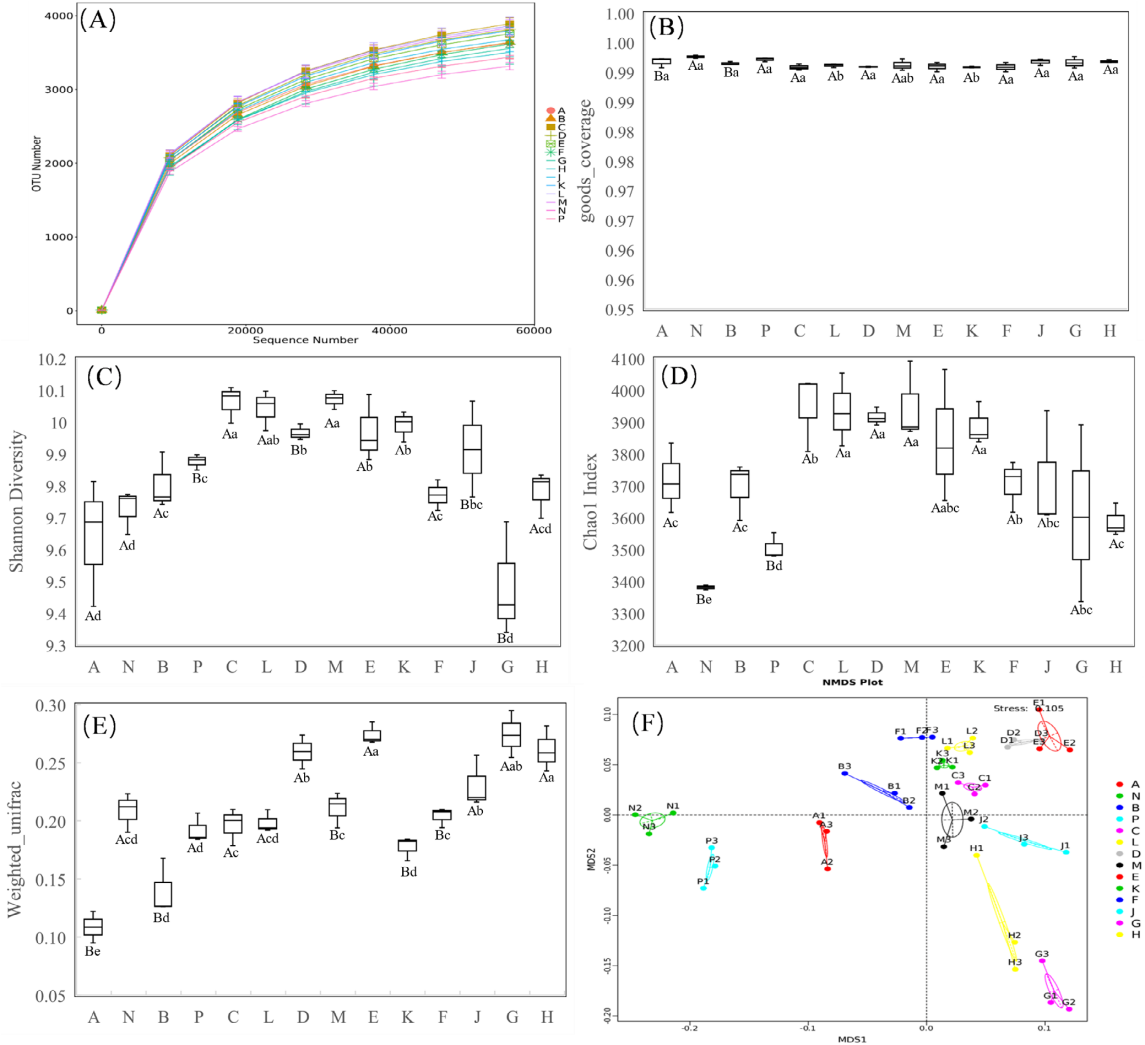
α and β diversity of soil bacterial communities at different altitudes and aspect in alpine meadows. A, B, C, D, E, F, and G represent gradients of 2800, 3000, 3200, 3400, 3600, 3800, and 4000 m above sea level on a shady aspect, respectively, and N, P, L, M, K, J and H represent gradients of 2800, 3000, 3200, 3400, 3600, 3800, and 4000 m above sea level on a sunny aspect, respectively. Same below. A, OUT number; B, goods_coverage; C, Shannon index; D, Chao1 index; E, β‐diversity (Weighted_unifrac); F, NMDS analysis. The lowercase letters indicate the difference between the same aspect and different altitudes (*p* ﹤ 0.05), and the capital letters indicate the difference between the same altitude and different aspect (*p* ﹤ 0.05).

Figure 2A,B illustrate that the number of bacterial operational taxonomic units (OTUs) in different treatments increased with the sequencing amount, resulting in total bacterial OTUs ranging from 3152 to 3874. The sequencing depth exceeded 0.995 in all cases, indicating adequate soil sample bacteria representation and data reliability. As depicted in Figure 2C, the Shannon index of soil bacteria initially increased and then decreased with increasing altitude, reaching its maximum value in the sunny aspect (10.07) at an altitude of 3400 m and in the shady aspect at an altitude of 3600 m (10.06). Except for 3200 m at the same altitude, the Shannon index in the sunny aspect was consistently higher than in the shady aspect. Similarly, the change in the Chao1 index of soil bacteria, shown in Figure 2D, mirrored that of the Shannon index.

Based on β‐diversity (Weighted_unifrac) and NMDS analysis (Figure 2E,F), it is evident that altitudes and aspects significantly influence the β‐diversity (Weighted‐unifrac) of soil bacterial communities, allowing for clear differentiation among different treatments. Consequently, soil bacterial community characteristics exhibit significant changes in response to altered altitudes and aspects.

### 3.2 Soil Bacterial Community Composition

**FIGURE 3:**
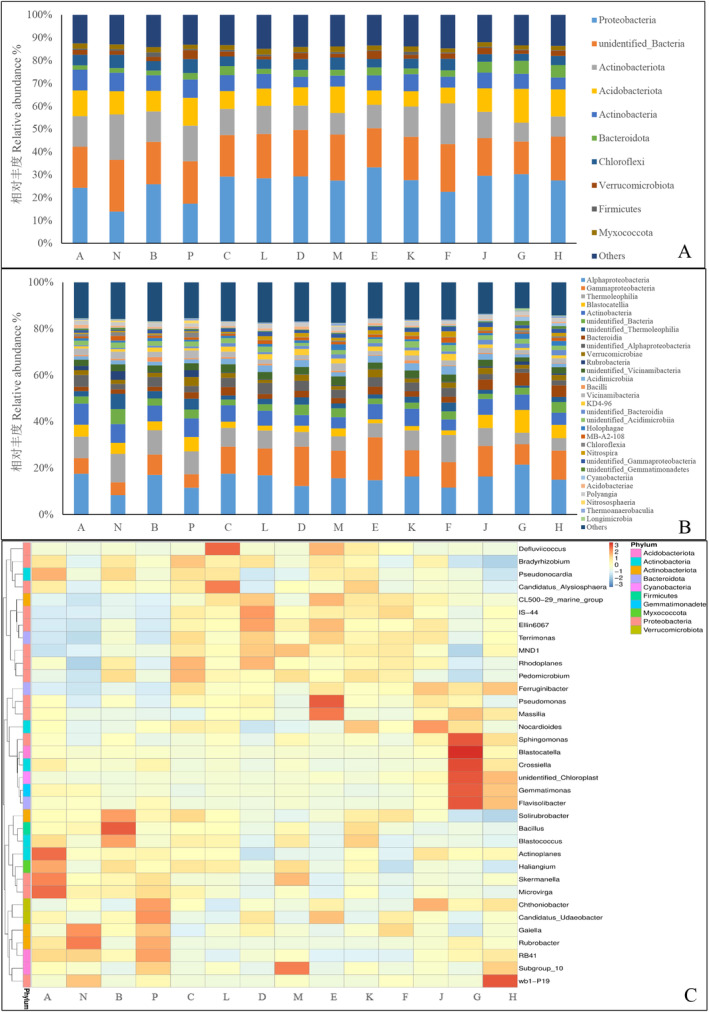
The composition of phylan, class and genera of soil bacterial at different altitudes and aspect in alpine meadows. A, Phylum level; B, class level; C, genus level.

From Figure 3A, the dominant soil bacterial communities at the phylum level in different treatments primarily consist of Proteobacteria, Actinobacteriota, Acidobacteriota, and Acidobacteria, with relative abundances of 22.20%, 12.56%, 9.61%, and 6.60%, respectively. Significant differences exist in the relative abundance of various bacterial phyla across different altitudes and aspects.

Figure 3 illustrates the dominant soil bacterial communities at the class level in various treatments of alpine meadow soils, mainly comprising *Alphaproteobacteria*, *Gammaproteobacteria*, and *Thermoleophilia*. Notably, there are significant differences in the relative abundance of different bacterial classes across altitudes and aspects. Furthermore, Figure 3C depicts the dominant soil bacterial communities at the genus level in various treatments of alpine meadow soils, including *Sphingomonas*, *Blastocatella*, and *RB41*. Similar to the findings at higher taxonomic levels, there are significant variations in the relative abundance of different bacterial genera across altitudes and aspects.

### 3.3 Linear Discriminant Analysis Effect Size (LefSe) and FAPROTAX Function

**FIGURE 4:**
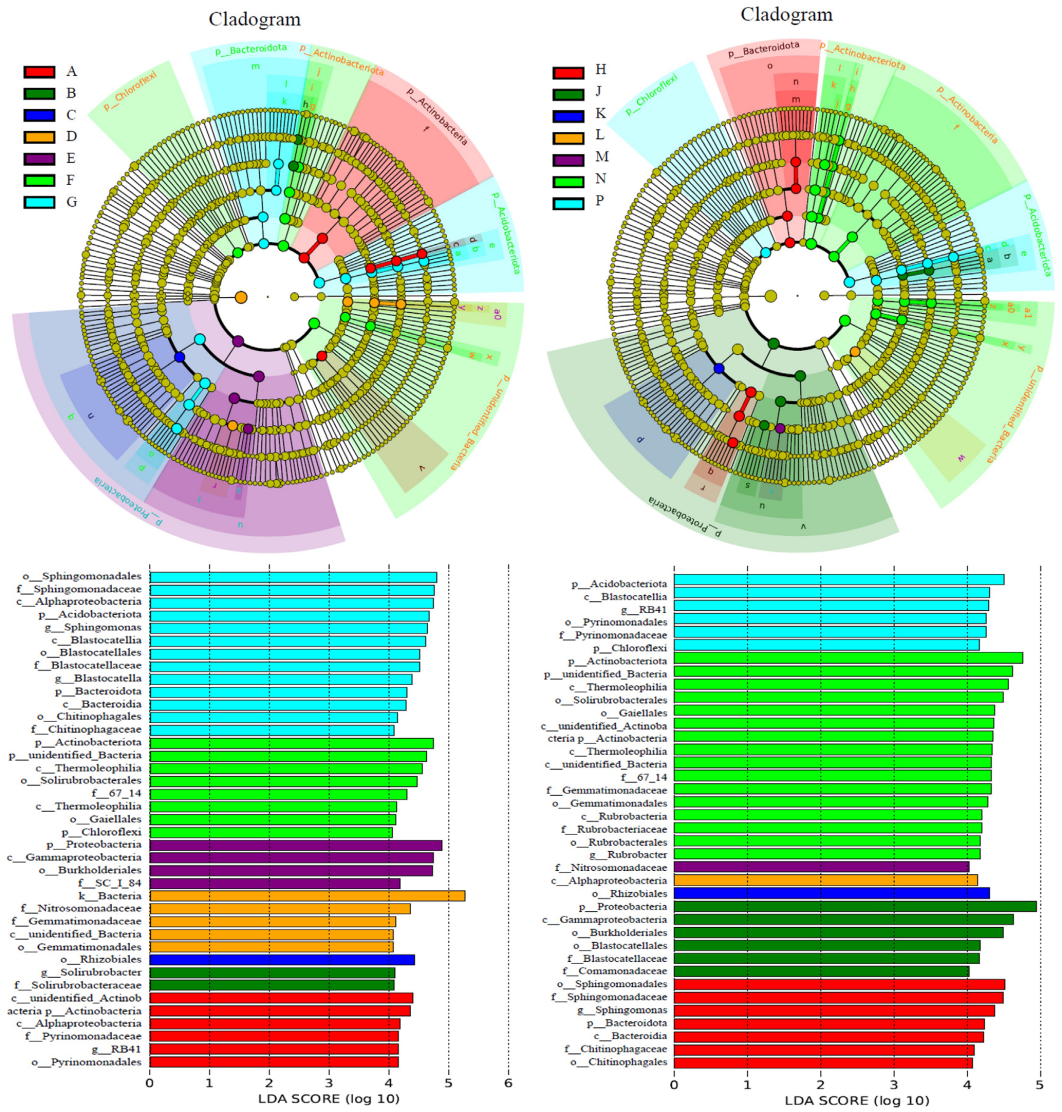
Structural differences of soil bacterial communities at different altitudes and aspects in alpine meadows.

From Figure 4, the LfSe revealed significant differences in bacterial community species across different altitudes and aspects. A total of 77 bacterial species exhibited variations at various taxonomic levels among the treatments of alpine meadows (with an LDA threshold of 4.0). Interestingly, the number of bacterial species showed an initial increase followed by a decrease with increasing altitude. Furthermore, within the exact altitudes, there were significant differences in the number of distinct species between shady and sunny aspects.

**FIGURE 5:**
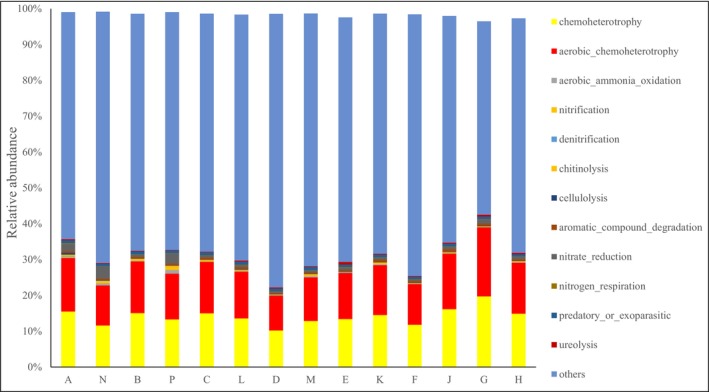
Analysis of soil bacterial nutrition function at different altitudes and aspects in alpine meadows.

According to the FAPROTAX analysis (Figure 5), 23.66%–46.02% of soil bacterial functions were annotated across different treatments of alpine meadows, falling short of 50%. The identified soil bacterial functions in alpine meadows included nine ecological functions, notably aerobic ammonia oxidation, nitrification, chitinolysis, cellulolysis, aerobic chemoheterotrophy, aromatic compound degradation, nitrate reduction, predatory or exoparasitic behavior, ureolysis, and chemoheterotrophy. Notably, the relative abundance of chemoheterotrophy and aerobic chemoheterotrophy exceeded 10%.

### 3.4 RDA and Main Effects Analysis

**FIGURE 6:**
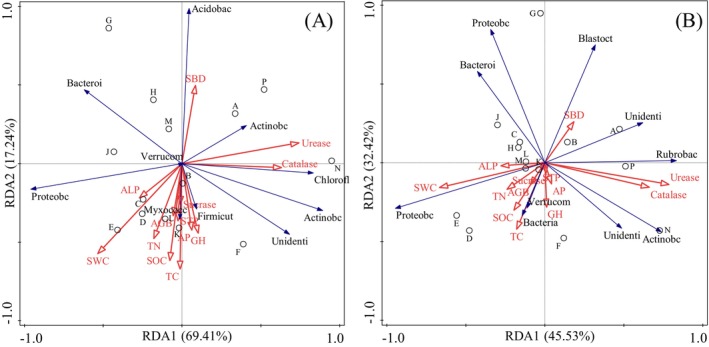
RDA analysis of bacterial phylum and class levels. A, Phylum level; B, Class level. red arrows represent independent variable, blue arrows represent dependent variable. Abbreviations: AGB, above‐ground biomass; AK, available potassium; ALP, alkaline phosphatase; AP, available phosphorus; GH, grass height; SBD, soil bulk density; SOC, soil organic carbon; STP, soil total porosity; SWC, soil water content; TC, total coverage; TN, total nitrogen.

The top 10 abundant soil bacterial phyla and classes and environmental factors such as vegetation and soil properties were selected for RDA. As depicted in Figure 6A, the first and second axes at the phylum level explained 69.41% and 17.24% of the environmental factors, respectively, totaling 86.65% of the explained variance. Similarly, Figure 6B illustrates that at the class level, the first and second axes explained 45.53% and 32.42% of the environmental factors, respectively, accounting for 77.95% of the total explained variance. The SWC and Urease significantly influenced on the composition of soil bacterial communities at the phylum level (Figure 6A). In contrast, SWC, Catalase and Urease significantly influenced the class‐level composition (Figure 6B). Specifically, SWC and Urease emerged as key environmental factors influencing soil bacterial communities.

The phyla and classes of dominant soil bacterial and diversity index were selected for Main effects analysis by SPSS 21.0. From Table 2, there were highly significant differences in the effect of altitude on soil bacterial composition, non‐significant differences in the effect of aspect orientation, and non‐significant differences in the effect of the altitude * aspect interaction.

**TABLE 2:** Significance tests of altitude and aspect on diversity and composition of bacterial community.

Treatment	Community diversity		Community composition	
	*F*	Significant	*F*	Significant
Altitude	8.047	0.000**	0.466	0.829
Aspect	0.613	0.438	0.400	0.531
Altitude * Aspect	1.476	0.210	0.457	0.836

*Note:* * means at the 0.05 level, ** means at the 0.01 level.

## 4 Discussion

Topographic factors are critical ecological determinants that influence biodiversity distribution patterns, with changes in habitat hydrothermal conditions driven by altitude and aspect orientation (Wang et al. 2022). The alpha diversity of soil bacterial communities is a direct indicator of changes in the quantity and richness of these communities (Chen et al. 2023). In our study, as altitude increased, the Shannon and Chao1 index of soil bacteria initially rose and then declined in sunny and shady aspects. Except for at 3200 m, the Shannon index was consistently higher in the sunny aspect compared to the shady aspect at the same altitude, aligning with findings from previous altitude‐related research (Liu et al. 2023). The variation in diversity characteristics of soil bacterial communities across different treatments was evident, with Shannon and Chao1 indices following altitude‐related patterns that mirrored variations in vegetation and soil nutrient characteristics. Utilizing Weighted Unifrac and NMDS analysis, our study effectively differentiated bacterial communities across different treatments, showcasing significant changes in soil bacterial community characteristics in response to altitude and aspect variations. As altitude and aspect change, they are often accompanied by shifts in various environmental factors, including climate, vegetation composition, and soil properties (Li et al. 2022a, 2022b), thereby prompting adjustments in bacterial community composition and diversity in response to these environmental conditions (Yuan et al. 2014; Liu et al. 2019; Liu, Yao, et al. 2019; Liu et al. 2020).

In alpine grassland ecosystems, bacteria, as the most numerous, diverse, and versatile taxa, play crucial roles in soil formation, decomposing organic matter, and nutrient cycling (Li et al. 2024; Chen et al. 2023). Understanding the altitudinal distribution pattern of soil bacteria in alpine meadows can enhance comprehension of soil bacterial community structures along environmental gradients, with significant ecological implications for exploiting microbial diversity resources in mountainous regions (Li et al. 2024). Previous studies, such as Liu et al. (2019), have identified *Proteobacteria*, *Actinobacteriota*, and *Acidobacteriota* as the main dominant phyla in surface soils of the Tibetan Plateau, with variations in abundance across different regions. Similarly, our research observed varied responses of soil bacteria to altitude and aspect across various taxonomic levels. At the phylum level, *Proteobacteria*, *Actinobacteriota*, *Acidobacteriota*, and *Acidobacteria* were predominant, with significant variability in relative abundance among different treatments. Since the Qilian Mountains are situated in the eastern region of the Tibetan Plateau, our research results are consistent with those of Liu, Cao, et al. 2019; Liu, Yao, et al. 2019). Proteobacteria and Actinobacteriota exhibit robust adaptive capacities and play pivotal roles in soil C and N cycles, with their relative abundance enhancing their resilience to extreme environments (Li et al. 2024). In our study, as altitude increased, the relative abundance of Proteobacteria in sunny and shady aspects initially increased and then decreased, with lower abundance observed in sunny aspects compared to shady aspects at the same altitude, consistent with soil nutrient and vegetation distributions. The relative abundances of *Actinobacteriota* and *Acidobacteria* varied across different altitudes and aspects, reflecting adaptation to environmental changes (Fang et al. 2018; Eichorst et al. 2018; Kirby 2005; Yergeau et al. 2010). At the class level, *Alphaproteobacteria*, *Gammaproteobacteria*, and *Thermoleophilia* dominated the soil bacterial community in alpine meadows across various altitudes and aspects, indicating changes in community structure due to altitude and aspect variations (Li et al. 2022a, 2022b). These changes in structure and function are interrelated, with each group performing distinct ecological functions (Li et al. 2022a, 2022b). As altitude and aspect shift, soil bacterial communities in alpine meadows adapt their composition and structure to accommodate environmental changes (Zhan et al. 2018).

Altitude and aspect alterations induce shifts in the vegetation community composition within alpine meadows by modifying water and heat conditions, consequently impacting the structure of bacterial communities, which, in turn, influences plant growth and development (Zhang et al. 2019). LEfSe analysis revealed substantial differences in soil bacterial community structure among different treatments, with varying bacterial species implicated in the metabolism of organic matter decomposition, energy flow, and C, N, and phosphorus cycling to validate this finding further. These bacterial communities play pivotal ecological roles in metabolic cycling, adapting to the heterogeneous distribution of grassland vegetation resources resulting from changes in altitude and aspect. FAPROTAX software analysis indicated that 23.66%–46.02% of soil bacteria functions were annotated across different alpine meadow treatments, with a predominant presence of nine ecological functional groups, each exceeding 10% relative abundance. Aerobic ammonia oxidation, nitrification, and chitinolysis were among the dominant functional groups.

The structure of the bacterial community is intricately linked to soil nutrient status and plant species (Li, Qiu, et al. 2021; Li, Yang, et al. 2021). Thus, exploring vegetation and soil conditions at an ecological scale holds significant scientific and practical importance. Jangid et al. (2011) demonstrated that the evolution of plant species influences changes in soil bacterial community structure, impacting the entire microbial habitat. Through RDA analysis, our research identified SWC and Urease as the primary factors influencing bacterial community composition, consistent with previous studies (Li et al. 2021; Jangid et al. 2011). However, our findings contrast with Li et al. (2024), who identified pH as a key environmental factor influencing soil bacterial community composition and diversity, along with soil alkaline dissolved N, TN, and organic C. These discrepancies likely stem from variations in geographic regions and vegetation types.

In the alpine meadow ecosystem, the changes observed in soil bacterial communities along altitude and aspect gradients are not simply responses to these factors. Still, they are also influenced by alterations in soil physicochemical properties induced by altitude and aspect variations. Consequently, the structure and diversity of soil bacterial communities in the East Qilian Mountains' alpine meadows reflect the combined influence of altitude, aspect orientation, and various soil physicochemical factors.

## 5 Conclusion

Altitudes and aspects orientation markedly alter the composition and diversity of soil bacterial communities. SWC and Urease emerged as key factors affecting the bacterial community. Consequently, it is imperative to integrate bacterial differences into the index system to evaluate the health of alpine grasslands.

## Author Contributions

**Qiang Li:** conceptualization (equal), data curation (equal), formal analysis (equal), funding acquisition (equal), methodology (equal), writing – original draft (equal), writing – review and editing (equal). **Ying Zhang:** data curation (equal), formal analysis (equal), methodology (equal), writing – review and editing (equal). **Xiaoni Liu:** data curation (equal), methodology (equal), writing – review and editing (equal). **Fan Yang:** data curation (supporting).

## Conflicts of Interest

The authors declare no conflicts of interest.

## Data Availability

All the raw data are uploaded to the database of NCBI SRA number PRJNA1110379 (https://www.ncbi.nlm.nih.gov/bioproject/PRJNA1110379).
